# Transcranial direct current stimulation and power spectral parameters: a tDCS/EEG co-registration study

**DOI:** 10.3389/fnhum.2014.00601

**Published:** 2014-08-07

**Authors:** Anna L. Mangia, Marco Pirini, Angelo Cappello

**Affiliations:** Department of Electrical, Electronic and Information Engineering, University of BolognaCesena, Italy

**Keywords:** tDCS, EEG, parietal cortex

## Abstract

Transcranial direct current stimulation (tDCS) delivers low electric currents to the brain through the scalp. Constant electric currents induce shifts in neuronal membrane excitability, resulting in secondary changes in cortical activity. Concomitant electroencephalography (EEG) monitoring during tDCS can provide valuable information on the tDCS mechanisms of action. This study examined the effects of anodal tDCS on spontaneous cortical activity in a resting brain to disclose possible modulation of spontaneous oscillatory brain activity. EEG activity was measured in ten healthy subjects during and after a session of anodal stimulation of the postero-parietal cortex to detect the tDCS-induced alterations. Changes in the theta, alpha, beta, and gamma power bands were investigated. Three main findings emerged: (1) an increase in theta band activity during the first minutes of stimulation; (2) an increase in alpha and beta power during and after stimulation; (3) a widespread activation in several brain regions.

## INTRODUCTION

The effects of a low electric current passing through the human scalp (transcranial direct current stimulation, tDCS) have been widely investigated (Paulus, 2011). Studies to date have focused on tDCS-induced modifications as advances in this field may further support the use of tDCS as a therapeutic tool for disorders characterized by electrophysiological and behavioral abnormalities. Electroencephalography (EEG) constitutes a simple and cost-effective methodology to measure modifications of brain activity during and after tDCS. EEG: (i) reflects the fluctuation of local field potentials resulting from the postsynaptic potentials of the cortical neurons, then the changes in neuronal resting membrane potential due to tDCS; (ii) identifies responses to tDCS within an area or across circuits, thereby aiding *in vivo* determination of the brain areas directly or indirectly affected by tDCS.

There are two combined EEG-tDCS methodologies: (i) the “oﬄine” method, with EEG recording performed after tDCS stimulation, to evaluate the short- and long-term after-effects induced by tDCS, and (ii) the “online” method, with EEG recording performed during tDCS stimulation, to evaluate the ongoing changes occurring during tDCS delivery. Several electrophysiological changes in EEG oscillations following tDCS have been observed using oﬄine methods in experiments involving a task for the subject or with the subject at rest. Ardolino et al. (2005) reported that cathodal stimulation of the motor cortex (1.5 mA, 35 cm^2^ electrode, 10 min) increased the power on delta and theta rhythms. Matsumoto et al. (2010) found that tDCS applied over the left primary motor area (1 mA, 35 cm^2^ electrode, 10 min) influenced event-related desynchronization (ERD) of the mu rhythm recorded during imaging of right hand grasping: mu ERD increased after anodal stimulation and decreased cathodal stimulation. These results are in partial disagreement with Ardolino et al.’s (2005) report, but the apparent discrepancy may be explained by the different state of the subject (rest state vs active state; Miniussi et al., 2012). Following anodal stimulation over the primary motor cortex, Polanía et al. (2012) reported that functional connectivity patterns significantly increased within the premotor, motor, and sensory motor areas of the stimulated hemisphere during motor activity. Notturno et al. (2013) investigated the effects of cathodal and anodal tDCS on primary motor cortex electric activity during a finger-tapping task. They found an increment of low alpha ERD in bilateral central, frontal areas and in the left inferior parietal region, as well as an increment of beta ERD in fronto-central and parieto-occipital regions after anodal stimulation compared to sham and cathodal stimulations. Finally, beta band coherence between signals from left sensorimotor cortex and activity of bilateral parietal, occipital, and right frontal regions were higher after anodal stimulation compared with the sham condition. Similarly, theta coherence between parietal and frontal regions was enhanced after anodal stimulation. Electrophysiological changes were also observed following stimulation over a non-motor area. Antal et al. (2004a) applied anodal and cathodal tDCS (1 mA, 35 cm^2^ electrode, 10 min) over Oz. They recorded EEG activity during the presentation of visual stimuli and found a decreased power in the beta and gamma frequency bands after cathodal stimulation, whereas no changes were observed after anodal stimulation. In a second study, Antal et al. (2004b) sought to establish if tDCS applied over the occipital cortex also affects visual-evoked potentials (VEPs). They found reversible excitability changes on the amplitude of the N70 and P100 component in a polarity-specific and time-specific way. On a working memory task, Keeser et al. (2011b) showed that 20 min of anodal stimulation (2 mA) over the left dorsolateral prefrontal cortex significantly reduced left frontal delta activity. Further, Zaehle et al. (2011a) stimulated the left dorsolateral prefrontal cortex during a working memory task and reported a significant reduction of power in the delta band after anodal stimulation. Lastly, Zaehle et al. (2011b) applied tDCS over the left temporal and left temporo-parietal cortex and investigated tDCS-induced effects on auditory evoked potentials after anodal, cathodal, and sham stimulation. They found that anodal tDCS over the temporal cortex increased auditory P50 amplitudes, while cathodal tDCS over the temporo-parietal cortex induced larger N1 amplitudes.

A few studies have also investigated changes in ongoing oscillatory brain activity subsequent to tDCS during rest (Ardolino et al., 2005; Zaehle et al., 2011b; Spitoni et al., 2013). Brain activity at rest constitutes an index of the internal state of the brain in the absence of an external input or motor output. Spitoni et al. (2013) studied the effect of tDCS on the postero-parietal cortex in the resting state, finding that anodal stimulation alters ongoing brain activity, specifically in the alpha band rhythm. These studies mostly focused on the “oﬄine” method for studying the effects of tDCS on EEG, whereas we think that the “online” approach is best for combined tDCS-EEG investigations. Indeed, (i) online approaches can yield information on the effects directly induced by tDCS, thus providing valuable information on tDCS mechanisms of action (this is particularly important to fully understand and exploit the potential of tDCS when used as a modulatory tool together with concomitant behavioral conditioning strategies, e.g., biofeedback; Bolognini et al., 2011; Wirth et al., 2011); (ii) EEG findings during tDCS can be interpreted as a surrogate marker for the effects of tDCS and thus can be used to optimize tDCS parameters in the context of a given application; (iii) online approaches could also be used for preventive treatment of neurological conditions characterized by abnormal peaks of cortical excitability, such as seizures (Faria et al., 2012; Schestatsky et al., 2013). Soekadar et al.’s (2014) study used the online approach to establish if learned EEG-based brain–machine interface control during tDCS is feasible. They recorded the learned desynchronization of murhythms (8–15 Hz) associated with motor imagery over C4 during anodal stimulation in a site placed 1 cm anterior to C4. They found a significant power increase in the lower frequencies mostly evident in the signal spectrum of the EEG channel closest to the stimulation electrode.

We present here a preliminary study aiming to: (i) investigate the effects of tDCS on spontaneous cortical activity at rest and (ii) differentiate between ongoing and after-effect modifications. To this end, we measured the modulation of spontaneous EEG during and after a session of anodal tDCS stimulation of the postero-parietal cortex. We focused solely on anodal stimulation (and sham control), excluding cathodal stimulation, since Spitoni et al. (2013) did not find significant EEG modifications after cathodal tDCS. Moreover, we concentrated on tDCS over the posterior parietal cortex since several studies demonstrated the utility of tDCS in the rehabilitation of the visual functions in both healthy subjects and patients with lesions on the parietal cortex, whereas few studies (Spitoni et al., 2013) investigated the ongoing electrophysiological effects of the stimulation.

We also aimed to determine the beginning and duration of tDCS-induced alterations. Several studies demonstrated that the effects of tDCS were stronger in the first 5 min after stimulation and persisted for about 20 min (Antal et al., 2010; Keeser et al., 2011b). Therefore, we studied the effects of tDCS over EEG power spectral parameters, specifically in theta, alpha, beta, and gamma bands, through a statistical analysis of variance, to determine: (1) the bands showing a change in their power, (2) the duration of the effects, and (3) their localization.

## MATERIALS AND METHODS

### SUBJECTS

Ten healthy subjects (seven males) participated in the study. Their ages ranged from 23 to 51 years. The inclusion criteria were (1) no history of neurological or psychiatric disorders, (2) no history of substance abuse or dependence, and (3) no use of medication affecting the central nervous system. All participants provided written consent to participate in the experiment. The study conformed to the Declaration of Helsinki and was approved by the local ethics committee.

### tDCS STIMULATION

We used the same protocol as Brunoni et al. (2012) and Spitoni et al. (2013). The Spitoni protocol aimed to investigate the electrophysiological changes induced through anodal and cathodal tDCS over posterior parietal areas during the resting state. A direct current of 1.5 mA (during stimulation the impedance value was maintained in a range 4–6 kΩ) induced through two saline-soaked surface sponge electrodes (7 × 4.5 cm) was delivered using a battery driven constant-current stimulator (neuroConn GmbH, Ehrenbergstr, Ilmenau, Germany). To avoid confounding biases that could have arisen from two electrodes with opposite polarities over the scalp, we used an extra-cephalic reference electrode for tDCS. The active electrode, the anode, was placed over the right posterior parietal cortex, and the reference electrode, the cathode, was placed over the ipsilateral deltoid muscle. The location of the active electrode was determined according to the 10–20 EEG standard montage, placing the electrode over P4, as suggested in previous studies (Spitoni et al., 2013). In the stimulation session, the current was ramped up from 0 to 1.5 mA in 30 s. Fifteen minutes after onset, the current was ramped down back to 0 in 30 s. Sham stimulation was used as control in the experiment to isolate the effects solely due to current stimulation and not due to the placebo and somatosensory effects that could arise from tDCS application. During the sham condition, the electrodes were located in the same positions as in the anodal stimulation, but the current was supplied only for the first 43 s (8 s ramp up, 30 s of DC stimulation and 5 s ramp down). This procedure ensured that the subjects felt the tingling sensation at the beginning of the stimulation.

### PROTOCOL

The subjects seated in a quiet room were asked verbally every 2 min to open or close their eyes, thus allowing us to conduct the subsequent analyses for two different behavioral conditions, the eyes open condition (EO) and the eyes closed condition (EC). During EO intervals, the subjects were instructed to fix a point in front of them and not to move their eyes. The participants did not know whether anodal tDCS or sham stimulation was delivered.

**FIGURE 1:**
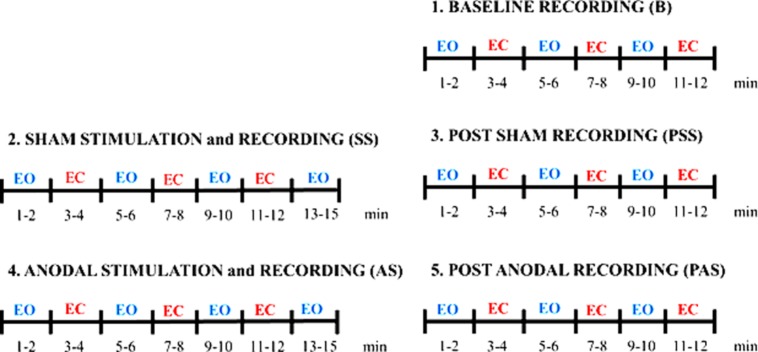
**Experimental protocol.** The protocol consists in a Baseline session (B), a Sham session (SS) and an Anodal session (AS) executed in sequence.

The protocol consists in a baseline session (B), a sham session (SS), and an anodal session (AS) executed in sequence, as shown in **Figure 1**. The sham session and an anodal session consisted each in a stimulation and recording session (SS and AS, respectively) and in a post recording session (PSS and PAS, respectively). It was decided to have the SS always before the AS to avoid the possible effects of AS on SS recordings. Given a choice, we hypothesized *a priori* the effects due to AS being equally or more relevant than the effects induced by SS. To further control for the placebo/somatosensory effects due to stimulation, we administered a side-effects questionnaire to the subjects to investigate any different perceptions during AS and SS.

### EEG RECORDING AND PREPROCESSING

Electroencephalography was recorded from 18 electrodes (Fp1, Fp2, F3, F4, F7, F8, Fz, C3, C4, Cz, P3, Pz, T3, T4, T5, T6, O1, and O2) positioned according to the international 10–20 layout using a Neurowave System (Khymeia, Italy). The EEG electrode over the stimulated area (P4) was removed from the registration cap to allow for the positioning of the stimulation electrode. EEG signals, referenced to linked ear lobes, were sampled at 512 samples/s, preliminarily band-pass filtered between 3 and 60 Hz. An additional stop-band filter at 50 Hz was applied (filters details are showed in **Table 1**). Trial datasets underwent (1) manual identification and rejection of artifactual segments; (2) decomposition in 2-s segments; (3) signal detrend by removing the mean and the linear trend in each 2-s segment (Muthuswamy and Thakor, 1998); (4) power spectral density (PSD) estimation for each 2-s segment (without overlap) through a modified periodogram method based on FFT-algorithm and Blackman Harris window. PSDs for each interval of interest (e.g., experimental sessions) were obtained by averaging the PSDs of the related two-second segments. Power values were extracted from the calculated PSDs in four frequency bands: theta (4–8 Hz), alpha (8–13 Hz), beta (13–25 Hz), and gamma (25–40 Hz).

**Table 1:** Band-pass and stop-band filters properties.

	Band-pass filter		Stop-band filter
	Low-pass	High-pass	
**Causality**	Causal	Causal	Causal
**Order**	Seventh	Second	Seventh
**Algorithm**	Elliptic	Elliptic	Elliptic
**Impulse response**	IIR	IIR	IIR

To compare the data of all subjects, we performed an intra-subject normalization by dividing the powers of each band, each electrode and each session (B, SS, PSS, AS, and PAS) by the corresponding power during the B session.

### STATISTICAL ANALYSIS

#### Effects of stimulation

We preliminarily tested for the normality of the power data distribution through a Kolmogorov–Smirnov test (Massey, 1951). We found a normal distribution of the data, justifying the subsequent use of ANOVA analyses.

A one-way ANOVA analysis on five levels (B, SS, PSS, AS, and PAS) was performed for each band and for each electrode using the data of all subjects, separately for the EOC and ECC. This analysis tested the hypothesis that there is a significant effect due to the stimulation conditions, against the general alternative that there is no significant effect. Since we were also interested in which pairs of conditions were significantly different, multiple comparison *post hoc* tests were also conducted when the ANOVA found a significant effect. We chose a significance level *p* = 0.01% and used the Bonferroni correction for multiple comparisons (Benjamini and Yekutieli, 2001). The correction factor was computed assuming independence between electrodes. Then this factor was given by the number of levels in the ANOVA analysis. For each electrode, the ANOVA test was carried out four times, once for each power band (theta, alpha, beta, and gamma). Then, the correction factor was 20 (5 levels × 4 bands). To further reorganize and interpret the results, we considered tDCS effects: (i) during SS and PSS (SHAM effects) as being significant only if SS and PSS power values were significantly different from B, and (ii) during AS and PAS sessions (STIMULATION effects) as being significant only if AS and PAS power values were significantly different from B, SS, and PSS simultaneously.

#### Effects over time

To investigate tDCS effects over time, AS, and PAS periods were divided into 2-min segments. For the EOC we obtained four segments for AS [AS1 (1–2 min), AS2 (5–6 min), AS3 (9–10 min), and AS4 (13–14 min)], and three segments for PAS [PAS1 (1–2 min), PAS2 (5–6 min), PAS3 (9–10 min)]. For the ECC we obtained three segments for AS [AS1 (3–4 min), AS2 (7–8 min), AS3 (11–12 min)], and three segments for PAS [PAS1 (3–4 min), PAS2 (7–8 min), PAS3 (11–12 min)].

Moreover, the effects of tDCS over time were analyzed by one way ANOVA analysis with ten levels for the EOC and one way ANOVA analysis with nine levels for the EC condition, with subsequent multiple comparison tests. For the EOC, the levels of the analysis were: B, SS, PSS, AS1, AS2, AS3, AS4, PAS1, PAS2, and PAS3. For the ECC, the levels of the analysis were: B, SS, PSS, AS1, AS2, AS3, PAS1, PAS2, and PAS3. For the analysis conducted to evaluate the stimulation effects, we chose a significance level *p* = 0.01% and used the Bonferroni corrections for multiple comparison tests (Benjamini and Yekutieli, 2001). In this case, the correction factor was 40 (10 levels × 4 bands) in EOC and 36 (9 levels × 4 bands) in ECC.

### SIDE-EFFECTS QUESTIONNAIRE

After the experiment, we administered a side-effects questionnaire to each subject to evaluate if there were differences in their physical perception of tDCS. If there were no differences between AS and SS we could exclude that tDCS effects over the EEG rhythm were due to the marked physical sensations associated with AS. The questionnaire consists in 11 questions on: tingling, itching sensation, burning sensation, pain, headache, fatigue, difficulty in concentrating, nervousness, visual perceptual changes, discomforting sensations, visual sensation associated with the start/end of the stimulation (Poreisz et al., 2007). The intensity of the side-effects were rated in a numerical discrete scale from 1–5, 1 being very mild and 5 being extremely strong for any given side-effect. To determine the statistical significance of each effect, side-effects were analyzed with an independent samples Mann–Whitney *U* test between the AS and SS conditions. We used the Mann–Whitney *U* test because the data distribution is not gaussian. For each side-effect, we computed the *p*-value and used a significance level *p* = 0.01.

### SOFTWARE TOOLS

MATLAB language and toolboxes were used for data processing and analysis (The Mathworks, USA). In particular, we used the Signal Processing Toolbox to preprocess the recorded data and the Statistics Toolbox for statistical analysis.

## RESULTS

### STATISTICAL ANALYSIS

#### Effects of stimulation

We analyzed the significance of each electrode-band couple for the different stimulation conditions as detailed in the “Materials and Methods.” We did not find any significant SHAM effect. The results reported below and in the next sessions refer only to the STIMULATION effects.

Considering the EOC, we did not find any significant effect. Considering the ECC, we found significant effects: (i) in theta band during AS for the electrodes F4, C4, O2, T4, T6, Cz, and Pz; (ii) in alpha band during AS for the electrodes C4, T6, Cz, and Pz; (iii) in alpha band during PAS for the electrodes Fp2, O2, F8, T4, T6, Fp1, F3, C3, P3, O1, T5, and Fz; (iv) in beta band during AS for the electrodes P3, O1, T3, T5, Cz, and Pz; (v) in beta band during PAS for the electrodes C3, P3, O1, T3, T5, and Fz.

**FIGURE 2:**
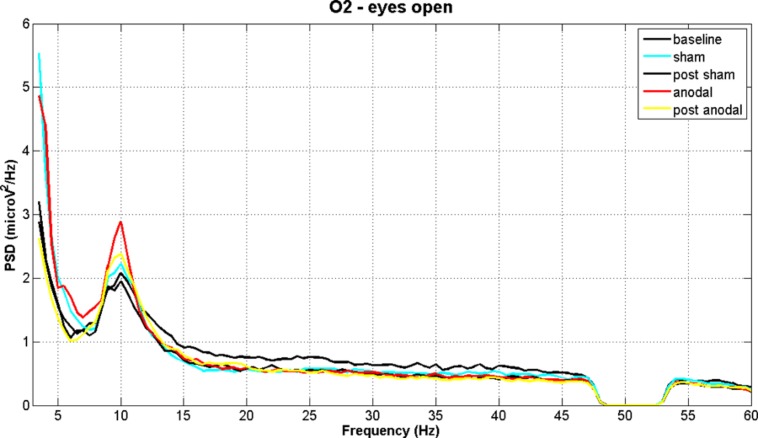
**EEG power spectral density of B, SS, PSS, AS, and PAS (EO condition) for electrode O2 (mean of all subjects).** Even if there are no significant effects considering the whole session duration, there is a significant peak increment in theta and alpha bands during stimulation. These effects are significant in the analysis of time effects.

**FIGURE 3:**
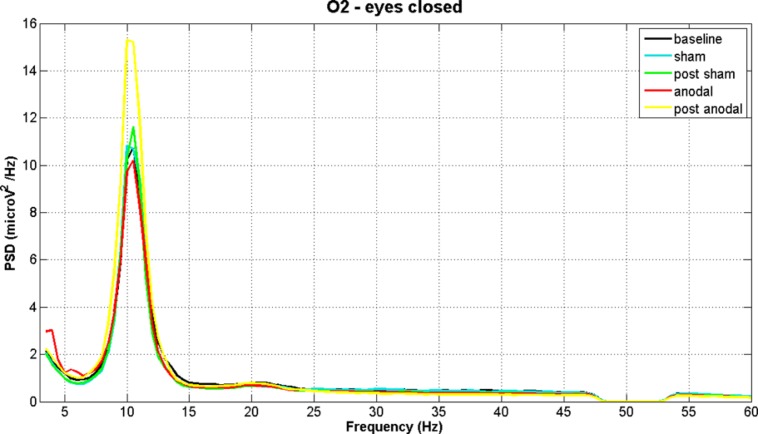
**EEG power spectral density of B, SS, PSS, AS, and PAS (EC condition) for electrode O2 (mean of all subjects).** The figure shows the increase in alpha band after stimulation.

**Figures 2** and **3** show PSD examples for electrode O2 in the five experimental conditions (B, SS, PSS, AS, PAS) for the EOC and ECC respectively.

#### Effects over time

**FIGURE 4:**
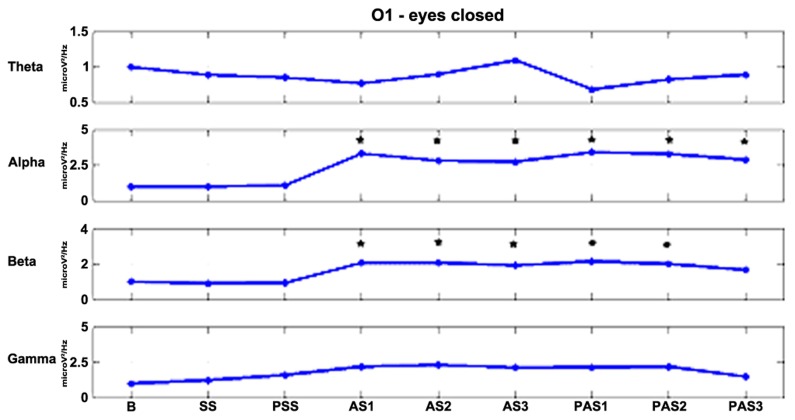
**The effects of stimulation over time for electrode O1 in the EC condition.** Alpha and beta powers increase significantly (*p < 0.01) during the first minutes of stimulation and the effects persist for at least 12 min after the end of stimulation in the alpha band and for 6 min in the beta band.

As described previously, we examined the effects of tDCS during 15 min of stimulation and for an additional 12 min after stimulation. In order to monitor the effects of stimulation in detail, the AS and PAS were divided into a certain number of 2-min epochs. **Tables 2** and **3** report the electrode-band couples with a significant activation during and after stimulation in the EOC and ECC, respectively. **Figure 4** show the power trend in the four bands analyzed for electrode O1 in the EC condition.

**Table 2:** Electrode-band couples with a significant activation during and after anodal tDCS in the eyes open condition.

Eyes open							
	AS1	AS2	AS3	AS4	PAS1	PAS2	PAS3
**Theta**	/	Fp2 Fp1 C4 O2 F8 F3 F7 Fz Cz Pz	/	/	/	Fp2 Fp1	Fp2 Fp1
**Alpha**	/	F4 C4 O2 Pz	C3 P3	/	/	/	/
**Beta**	/	/	/	/	/	/	/
**Gamma**	/	/	/	/	/	/	/

**Table 3:** Electrode-band couples with a significant activation during and after anodal tDCS in the eyes closed condition.

Eyes closed							
	AS1	AS2	AS3	PAS1	PAS2	PAS3
**Theta**	T4 Cz	C4	F4 C4 Fz Pz	T4	Fp2 T4 Fp1 Fz	Fp1 F3 Fz
**Alpha**	O2 C3 P3 O1 T5 Cz	C4 O2 P3 O1 Cz Pz	C4 O2 C3 P3 O1 Pz	O2 Fp1 F3 C3 P3 O1 T5 Fz Pz	O2 Fp1 F3 C3 P3 O1 T5 Fz Pz	O2 F3 C3 P3 O1 T5 Fz
**Beta**	O1 P3 Cz	O1 P3 C3 Cz	O1 P3 C3 Pz	O1 P3 C3	O1 P3 C3	C3 P3
**Gamma**	/	/	/	/	/	/

### SIDE-EFFECTS QUESTIONNAIRE

The results of the Mann–Whitney *U* test demonstrated that none of the 11 side-effects showed a significant difference between the two conditions of SS and AS stimulation, thereby excluding that the effects on the EEG rhythm are due to the physical perception of the stimulation.

## DISCUSSION

### STATISTICAL ANALYSIS

#### Effects of stimulation

The mechanism underlying the neuromodulatory effects induced by tDCS is highly controversial and numerous studies have addressed the topic. Several experimental studies have suggested that neurons respond to tDCS-induced membrane polarization changes (Liebetanz et al., 2002), thereby leading to a reduction in spontaneous neuronal firing rates after cathodal tDCS and an opposite effect after anodal stimulation. Accordingly, the first study performed on the motor cortex showed that cathodal polarization strongly inhibited motor cortex excitability, whereas anodal polarization increased motor cortex excitability (Nitsche and Paulus, 2000). In the light of these findings, we expected to find a decrease of theta and alpha powers associated with cortical deactivation, and an increase in beta and gamma powers associated with cortical activation. On the contrary, our main findings are the increase in theta power during tDCS and the increase in alpha and beta powers during and after stimulation in the EC condition. We found no effect in the EOC. This finding can be explained by the fact that tDCS disrupts the equilibrium on both excitatory and inhibitory neurons inducing an increase in theta band activity in the first minutes of stimulation and in alpha and beta bands during and after tDCS. The increase in alpha band after anodal stimulation confirms the results of Spitoni et al. (2013).

#### Effects over time

The effect of tDCS over time is a critical issue because the after-effect of stimulation might last from minutes to hours, depending on the intensity and duration of the stimulation. Antal et al. (2010) found that at equal intensity and duration the effects of stimulation lasted longer on the motor cortex than the posterior cortex. We observed significant effects both during and after tDCS. In the EOC the effect was predominant during AS2 in theta and alpha bands, while in the ECC it was present throughout stimulation and the whole interval after stimulation. In particular, theta power had a significant activation in the centro-parietal regions during stimulation and a propagation in the frontal region after stimulation. In the EOC, a significant activation was found mainly in the frontal region. These results are consistent with the spontaneous flow of information between sources of brain activity in the theta band. Indeed, Michels et al. (2013) found that in resting state condition an information flow involves both parietal and frontal regions in the ECC, but only the frontal region in the EOC.

Alpha power increased significantly during and after tDCS. The effects persisted for all 12 min after stimulation and we did not observe a decline of these effects. Alpha amplitude modulation was observed in both posterior and frontal regions. Several studies demonstrated that tDCS increases the coherence of the cerebral rhythm and the interaction between inter- and intra-cerebral cortexes (Keeser et al., 2011a; Hampstead et al., 2013). Polanía et al. (2012) demonstrated that tDCS applied over the primary motor cortex produces modifications in EEG synchronization and functional organization in healthy subjects. Furthermore, they demonstrated a coherence modification in all frequency bands (theta, alpha, beta, and gamma).

We found an increase in beta band power during and after stimulation for three electrodes positioned in the contralateral site with respect to the site of tDCS. We did not find significant effects in the gamma band.

#### Comparison between EC and EO responsiveness to tDCS stimulation

We found clear-cut differences between the EOC and ECC in terms of responsiveness to tDCS, with the EEG power parameters much more sensitive to tDCS stimulation in the ECC than in the EOC. Several studies investigated the differences in EEG signal properties between the ECC and the EOC in resting state. Barry et al.’s (2007) study showed that: (i) the signal power is lower in the EOC than in the ECC for the delta, theta and alpha bands; (ii) lateral frontal delta, posterior theta and right-posterior beta are reduced, while the left-frontal beta powers are increased in EOC compared to ECC; (iii) no significant topographic differences were evident for the power in the alpha band between the two conditions. These results allow us to interpret our findings in terms of the different processing capability of the brain in the two conditions. In particular, the brain is much more stimulated in the EOC than in the ECC: the lower powers in delta, theta and alpha bands in the EOC reported by Barry et al. (2007) are clearly related to a lower involvement of cortico-thalamic (idling) dynamics, and (possibly) to a higher involvement of intra-cortical (processing) dynamics. This is further confirmed by the higher value of frontal beta power in the EOC. Given the above premises, the higher responsiveness to tDCS in the ECC could be interpreted in general terms as the consequence of a higher processing capability to the external tDCS stimuli available in the ECC than in the EOC.

Comparing the results of studies on tDCS-induced EEG modifications induced by tDCS in resting state conditions, we found that stimulation of the posterior cortex primarily generates changes in alpha power (our results and Spitoni et al., 2013). This is not surprising since it is well known that the alpha rhythm is mainly generated in the posterior cortex and that posterior cortexes resonate to external stimulations in the alpha band. (Sadato et al., 1998; Laufs et al., 2003; Tyvaert et al., 2008; Omata et al., 2013). Several other studies showed that stimulating other areas would produce power changes in different bands, e.g., stimulation of the prefrontal cortex induced a significant decrease in frontal delta power (Ardolino et al., 2005; Keeser et al., 2011b) and a significant increase in frontal beta power (Keeser et al., 2011b) in resting state condition.

Plainly, the significant effects found in our study are not easy to interpret. Even taking into account the simplest interpretation of EEG rhythms, that power in delta, theta, and alpha bands is positively correlated with cortical idling, and power in beta and gamma bands is positively correlated with cortical processing (Başar et al., 2001; Barry et al., 2007), the effects found need to be further investigated with more complex interpretative tools. Future investigations on the results of combined tDCS–EEG experiments could benefit from the interpretative power of neural mass models. To date, these models have been successfully used to interpret EEG power modifications as dynamic modifications of cortical network functional connectivity during sleep rhythms (Cona et al., 2014), due to cognitive and motor tasks (Cona et al., 2009), and, intriguingly, dynamic perturbation of brain networks with transcranial magnetic stimulation (Cona et al., 2011), a companion technology to tDCS.

## CONCLUSION

This study investigated the ongoing and after-effects of anodal tDCS applied over the postero-parietal cortex in a resting brain. We compared the power spectral parameters obtained from a sham condition (during and post) and a real condition (during and post) of stimulation. We found that the main effect regards the theta, alpha, and beta bands beginning with the start of stimulation and lasting at least 12 min after the end of tDCS. We confirmed the results of Spitoni et al. (2013), the only study to investigate the effects of tDCS over the parietal cortex in resting state, and we also analyzed the effects during stimulation. Possible future developments should aim to reach a broader interpretation of current results.

## Conflict of Interest Statement

The authors declare that the research was conducted in the absence of any commercial or financial relationships that could be construed as a potential conflict of interest.
