# Supplementary material for: Transcranial direct current stimulation and power spectral parameters: a tDCS/EEG co-registration study
Source: Front Hum Neurosci. 2014 Aug 7;8:601. doi: 10.3389/fnhum.2014.00601 (PMC4124721; doi:10.3389/fnhum.2014.00601)
Supplement: Supplementary file 1 [file Data_Sheet_1.DOCX]

***Supplementary Material***

**Transcranial Direct Current Stimulation and Power Spectral Parameters: a tDCS/EEG co-registration study**

**Anna Lisa Mangia^1^*, Marco Pirini^1^, Angelo Cappello^1^**

^1^ Department of Electrical, Electronic and Information Engineering (DEI), University of Bologna, Cesena, Italy

*** Correspondence:** Corresponding Author, Department of Electrical, Electronic and Information Engineering (DEI), University of Bologna, Cesena, 47521, Italy.

[annalisa.mangia2@unibo.it](mailto:annalisa.mangia2@unibo.it)

Table 1: The table shows the p-value, the F-value and the significance of each couple band-electrode in EO condition in the comparison between B, SS, PSS, AS and PAS1 (stimulation-effects). The significance (s) is reported in three conditiosn: (*) indicates the significance without Bonferroni correction (p<0.01), (**) indicates the significance after the Bonferroni correction (p<0.01/20), (***) indicate the significance, after the Bonferroni correction, of the AS and PAS condition with respect the B, SS and PSS (p<0.01/20).

| **EYES OPEN** | | | | | | | | | | | | |
| --- | --- | --- | --- | --- | --- | --- | --- | --- | --- | --- | --- | --- |
|  | theta | | | alpha | | | beta | | | gamma | | |
|  | p-value | F-value | sign | p-value | F-value | sign | p-value | F-value | sign | p-value | F-value | sign |
| Fp2 | 0,00000 | 9,91648 | (**) | 0,06547 | 2,23549 |  | 0,20967 | 1,47599 |  | 0,01011 | 3,38170 |  |
| F4 | 0,49692 | 0,84623 |  | 0,49743 | 0,84540 |  | 0,29622 | 1,23512 |  | 0,32997 | 1,15743 |  |
| C4 | 0,47077 | 0,88926 |  | 0,41338 | 0,98999 |  | 0,39195 | 1,03028 |  | 0,31262 | 1,19649 |  |
| O2 | 0,36931 | 1,07473 |  | 0,22725 | 1,42076 |  | 0,11593 | 1,86989 |  | 0,07149 | 2,17992 |  |
| F8 | 0,46539 | 0,89833 |  | 0,15974 | 1,65920 |  | 0,00133 | 4,58968 | (*) | 0,01458 | 3,16095 |  |
| T4 | 0,05183 | 2,38228 |  | 0,00001 | 7,57855 | (**) | 0,00404 | 3,93071 | (*) | 0,70567 | 0,54110 |  |
| T6 | 0,65399 | 0,61247 |  | 0,57252 | 0,72942 |  | 0,54438 | 0,77175 |  | 0,00013 | 5,95445 | (**) |
| Fp1 | 0,00000 | 10,59812 | (**) | 0,01190 | 3,28382 |  | 0,03912 | 2,55724 |  | 0,00231 | 4,26260 | (*) |
| F3 | 0,90956 | 0,24993 |  | 0,10577 | 1,92934 |  | 0,18059 | 1,57711 |  | 0,24802 | 1,36022 |  |
| C3 | 0,07695 | 2,13322 |  | 0,00019 | 5,74426 | (**) | 0,00195 | 4,36276 | (*) | 0,15093 | 1,69685 |  |
| P3 | 0,00718 | 3,58712 |  | 0,00000 | 8,28532 | (**) | 0,00385 | 3,95915 | (*) | 0,01824 | 3,02507 |  |
| O1 | 0,00186 | 4,39057 |  | 0,00028 | 5,49984 | (**) | 0,00171 | 4,44294 | (*) | 0,00235 | 4,25310 | (*) |
| F7 | 0,72168 | 0,51922 |  | 0,19875 | 1,51239 |  | 0,04132 | 2,52337 |  | 0,12265 | 1,83319 |  |
| T3 | 0,00058 | 5,08478 |  | 0,07316 | 2,16528 |  | 0,00741 | 3,56888 | (*) | 0,13449 | 1,77290 |  |
| T5 | 0,00031 | 5,44081 | (**) | 0,00046 | 5,21460 | (**) | 0,00577 | 3,71806 | (*) | 0,08864 | 2,04300 |  |
| Fz | 0,15287 | 1,68840 |  | 0,07816 | 2,12327 |  | 0,15069 | 1,69791 |  | 0,01141 | 3,30910 |  |
| Cz | 0,11430 | 1,87912 |  | 0,00932 | 3,43073 | (*) | 0,11629 | 1,86791 |  | 0,07366 | 2,16098 |  |
| Pz | 0,08403 | 2,07717 |  | 0,00012 | 6,02821 | (**) | 0,00001 | 7,24642 | (**) | 0,00028 | 5,50814 | (**) |

Table 2: The table shows the p-value, the F-value and the significance of each couple band-electrode in EC condition in the comparison between B, SS, PSS, AS and PAS1 (stimulation-effects). The significance (s) is reported in three conditiosn: (*) indicates the significance without Bonferroni correction (p<0.01), (**) indicates the significance after the Bonferroni correction (p<0.01/20), (***) indicate the significance, after the Bonferroni correction, of the AS and PAS condition with respect the B, SS and PSS (p<0.01/20).

| **EYES CLOSE** | | | | | | | | | | | | |
| --- | --- | --- | --- | --- | --- | --- | --- | --- | --- | --- | --- | --- |
|  | theta | | | alpha | | | beta | | | gamma | | |
|  | p-value | F-value | s | p-value | F-value | sign | p-value | F-value | sign | p-value | F-value | s |
| Fp2 | 0,00003 | 6,87943 | (**) | 0,00003 | 6,83202 | (***) | 0,59849 | 0,69150 |  | 0,47445 | 0,88351 |  |
| F4 | 0,00007 | 5,03744 | (***) | 0,01315 | 3,23384 |  | 0,00823 | 3,51769 | (*) | 0,00022 | 5,69386 | (**) |
| C4 | 0,00000 | 13,66868 | (***) | 0,00000 | 9,77080 | (***) | 0,00000 | 10,23386 | (**) | 0,00002 | 7,22553 | (**) |
| O2 | 0,00400 | 3,95220 | (***) | 0,00000 | 27,65414 | (***) | 0,00000 | 12,29829 | (**) | 0,00513 | 3,80309 |  |
| F8 | 0,26820 | 1,30674 |  | 0,00000 | 11,81246 | (***) | 0,00001 | 7,43366 | (**) | 0,35595 | 1,10276 |  |
| T4 | 0,00000 | 27,06526 | (***) | 0,00000 | 15,29106 | (***) | 0,00493 | 3,82670 | (*) | 0,77630 | 0,44457 |  |
| T6 | 0,00009 | 6,22067 | (***) | 0,00003 | 6,20693 | (***) | 0,00090 | 4,84810 | (*) | 0,00312 | 4,10123 |  |
| Fp1 | 0,00000 | 10,13106 | (**) | 0,00000 | 9,84762 | (***) | 0,50029 | 0,84113 |  | 0,59534 | 0,69609 |  |
| F3 | 0,00001 | 7,45191 | (**) | 0,00000 | 14,41640 | (***) | 0,00001 | 7,38426 |  | 0,31154 | 1,19996 |  |
| C3 | 0,01735 | 3,06451 |  | 0,00000 | 23,50859 | (***) | 0,00000 | 18,68782 | (***) | 0,31329 | 1,19593 |  |
| P3 | 0,50498 | 0,83359 |  | 0,00000 | 38,97035 | (***) | 0,00000 | 26,02585 | (***) | 0,35938 | 1,09567 |  |
| O1 | 0,17278 | 1,60887 |  | 0,00000 | 29,18315 | (***) | 0,00000 | 18,95819 | (***) | 0,00125 | 4,64681 |  |
| F7 | 0,40799 | 1,00057 |  | 0,02781 | 2,77501 |  | 0,16136 | 1,65470 |  | 0,30924 | 1,20530 |  |
| T3 | 0,00034 | 5,42699 | (**) | 0,26539 | 1,31416 |  | 0,00043 | 5,27972 | (***) | 0,01482 | 3,16084 |  |
| T5 | 0,15177 | 1,69555 |  | 0,00000 | 18,15574 | (***) | 0,00000 | 13,36395 | (***) | 0,08062 | 2,10750 |  |
| Fz | 0,00000 | 14,30752 | (**) | 0,00000 | 21,15472 | (***) | 0,00007 | 6,39110 | (***) | 0,50943 | 0,82649 |  |
| Cz | 0,00003 | 6,82041 | (***) | 0,00000 | 10,93108 | (***) | 0,00000 | 10,48662 | (***) | 0,00246 | 4,24379 |  |
| Pz | 0,00035 | 5,41567 | (***) | 0,00000 | 17,24579 | (***) | 0,00000 | 8,96042 | (***) | 0,00186 | 4,41008 |  |

Table 3: The table shows the p-value, the F-value and the significance of each couple band-electrode in EO condition in the comparison between B, SS, PSS, AS1, AS2, AS3, AS4, PAS1, PAS2 and PA3 (time-effects). The significance (s) is reported in three conditiosn: (*) indicates the significance without Bonferroni correction (p<0.01), (**) indicates the significance after the Bonferroni correction (p<0.01/40), (***) indicate the significance, after the Bonferroni correction, of the AS and PAS condition with respect the B, SS and PSS (p<0.01/40).

| **EYES OPEN** | | | | | | | | | | | | |
| --- | --- | --- | --- | --- | --- | --- | --- | --- | --- | --- | --- | --- |
|  | theta | | | alpha | | | beta | | | gamma | | |
|  | p-value | F-value | s | p-value | F-value | s | p-value | F-value | s | p-value | F-value | s |
| Fp2 | 0,00000 | 13,48195 | (***) | 0,12543 | 1,56595 |  | 0,57698 | 0,84312 |  | 0,06257 | 1,83256 |  |
| F4 | 0,04720 | 1,93586 |  | 0,00006 | 5,85947 | (***) | 0,04782 | 1,93114 |  | 0,04687 | 1,93839 |  |
| C4 | 0,00004 | 4,97342 | (***) | 0,00004 | 4,95482 | (***) | 0,04814 | 1,92865 |  | 0,04973 | 1,91691 |  |
| O2 | 0,00005 | 4,91615 | (***) | 0,00005 | 6,89704 | (***) | 0,06398 | 1,82432 |  | 0,07185 | 1,78100 |  |
| F8 | 0,00000 | 7,51130 | (***) | 0,06191 | 1,83648 |  | 0,02900 | 2,10952 |  | 0,18027 | 1,41766 |  |
| T4 | 0,00565 | 2,66329 | (*) | 0,00042 | 3,49031 | (*) | 0,00398 | 2,77786 | (*) | 0,02142 | 2,21505 |  |
| T6 | 0,17366 | 1,43332 |  | 0,08192 | 1,73150 |  | 0,09437 | 1,67741 |  | 0,00339 | 2,83001 | (*) |
| Fp1 | 0,00000 | 15,03025 | (***) | 0,03714 | 2,02203 |  | 0,24799 | 1,27941 |  | 0,03455 | 2,04775 |  |
| F3 | 0,00000 | 5,19098 | (***) | 0,27635 | 1,23028 |  | 0,52939 | 0,89590 |  | 0,57824 | 0,84174 |  |
| C3 | 0,00041 | 3,49846 | (*) | 0,00024 | 3,66250 | (***) | 0,02294 | 2,19134 |  | 0,50776 | 0,92041 |  |
| P3 | 0,00003 | 4,30247 | (*) | 0,00000 | 5,31510 | (***) | 0,03621 | 2,03107 |  | 0,14040 | 1,52070 |  |
| O1 | 0,00010 | 3,92985 | (**) | 0,00074 | 3,31479 | (*) | 0,01095 | 2,44353 |  | 0,02586 | 2,14971 |  |
| F7 | 0,00001 | 4,68154 | (***) | 0,56226 | 0,85930 |  | 0,30173 | 1,18942 |  | 0,49930 | 0,93012 |  |
| T3 | 0,00056 | 3,40535 | (*) | 0,23141 | 1,31017 |  | 0,04512 | 1,95212 |  | 0,17465 | 1,43094 |  |
| T5 | 0,00003 | 4,35848 | (**) | 0,00055 | 3,40845 | (*) | 0,04797 | 1,92994 |  | 0,18777 | 1,40044 |  |
| Fz | 0,00000 | 7,56427 | (***) | 0,15531 | 1,47957 |  | 0,52196 | 0,90427 |  | 0,11537 | 1,59911 |  |
| Cz | 0,00004 | 4,18541 | (***) | 0,02884 | 2,11146 |  | 0,37477 | 1,08394 |  | 0,33442 | 1,14028 |  |
| Pz | 0,00003 | 4,32360 | (***) | 0,00000 | 5,78812 | (***) | 0,00000 | 4,89278 | (**) | 0,00060 | 3,37961 | (*) |

Table 4: The table shows the p-value, the F-value and the significance of each couple band-electrode in EC condition in the comparison between B, SS, PSS, AS1, AS2, AS3, AS4, PAS1, PAS2 and PAS3 (time-effects). The significance (s) is reported in three conditions: (*) indicates the significance without Bonferroni correction (p<0.01), (**) indicates the significance after the Bonferroni correction (p<0.01/36), (**) indicate the significance, after the Bonferroni correction, of the AS and PAS condition with respect the B, SS and PSS (p<0.01/36).

| **EYES CLOSE** | | | | | | | | | | | | | |
| --- | --- | --- | --- | --- | --- | --- | --- | --- | --- | --- | --- | --- | --- |
|  | theta | | | alpha | | | beta | | | gamma | | | |
|  | p-value | F-value | sign | p-value | F-value | sign | p-value | F-value | sign | p-value | F-value | | sign |
| Fp2 | 0,00017 | 4,02145 | (***) | 0,00070 | 3,53366 | (*) | 0,74541 | 0,63794 |  | 0,78021 | 0,59667 |  | |
| F4 | 0,00003 | 4,03875 | (***) | 0,12223 | 1,61191 |  | 0,06726 | 1,86008 |  | 0,00459 | 2,87190 | (*) | |
| C4 | 0,00000 | 8,70197 | (***) | 0,00001 | 5,09023 | (***) | 0,00000 | 6,13439 | (**) | 0,00002 | 4,76163 | (**) | |
| O2 | 0,00469 | 2,86389 | (*) | 0,00000 | 14,15470 | (***) | 0,00000 | 6,33374 | (**) | 0,04323 | 2,03594 |  | |
| F8 | 0,43362 | 1,00414 |  | 0,00000 | 5,86189 | (**) | 0,00015 | 4,07554 | (**) | 0,37155 | 1,08907 |  | |
| T4 | 0,00000 | 13,51774 | (***) | 0,00000 | 7,60158 | (**) | 0,00114 | 3,36601 | (*) | 0,02506 | 2,24630 |  | |
| T6 | 0,00120 | 3,34641 | (*) | 0,51521 | 0,90236 |  | 0,00695 | 2,72185 | (*) | 0,02054 | 2,32152 |  | |
| Fp1 | 0,00000 | 5,57060 | (***) | 0,00001 | 4,97888 | (***) | 0,67057 | 0,72363 |  | 0,78040 | 0,59644 |  | |
| F3 | 0,00001 | 5,23098 | (***) | 0,00000 | 7,25957 | (***) | 0,00014 | 4,08870 | (**) | 0,51608 | 0,90131 |  | |
| C3 | 0,00294 | 3,03057 | (*) | 0,00000 | 11,80555 | (***) | 0,00000 | 9,68550 | (***) | 0,56631 | 0,84226 |  | |
| P3 | 0,09267 | 1,72873 |  | 0,00000 | 19,69973 | (***) | 0,00000 | 13,29208 | (***) | 0,61844 | 0,78261 |  | |
| O1 | 0,09207 | 1,73145 |  | 0,00000 | 14,81725 | (***) | 0,00000 | 9,78090 | (***) | 0,01060 | 2,56754 |  | |
| F7 | 0,20910 | 1,37327 |  | 0,17228 | 1,46157 |  | 0,28883 | 1,21858 |  | 0,44058 | 0,99508 |  | |
| T3 | 0,00052 | 3,64162 | (*) | 0,66536 | 0,72952 |  | 0,00136 | 3,30217 | (*) | 0,01768 | 2,37775 |  | |
| T5 | 0,08900 | 1,74553 |  | 0,00000 | 9,17247 | (***) | 0,00000 | 6,98598 | (**) | 0,30541 | 1,19066 |  | |
| Fz | 0,00000 | 9,28820 | (***) | 0,00000 | 10,51237 | (***) | 0,00056 | 3,61232 | (*) | 0,75177 | 0,63050 |  | |
| Cz | 0,00009 | 4,44606 | (***) | 0,00000 | 5,66459 | (***) | 0,00000 | 5,50613 | (***) | 0,02023 | 2,32719 |  | |
| Pz | 0,00000 | 6,24298 | (***) | 0,00000 | 11,57693 | (***) | 0,00000 | 6,93207 | (***) | 0,00042 | 3,71499 | (*) | |
